# Webcrawling and machine learning as a new approach for the spatial distribution of atmospheric emissions

**DOI:** 10.1371/journal.pone.0200650

**Published:** 2018-07-16

**Authors:** Susana Lopez-Aparicio, Henrik Grythe, Matthias Vogt, Matthew Pierce, Islen Vallejo

**Affiliations:** 1 NILU - Norwegian Institute for Air Research, Kjeller, Norway; 2 University of Helsinki, Helsinki, Finland; Universidade de Vigo, SPAIN

## Abstract

In this study we apply two methods for data collection that are relatively new in the field of atmospheric science. The two developed methods are designed to collect essential geo-localized information to be used as input data for a high resolution emission inventory for residential wood combustion (RWC). The first method is a webcrawler that extracts openly online available real estate data in a systematic way, and thereafter structures them for analysis. The webcrawler reads online Norwegian real estate advertisements and it collects the geo-position of the dwellings. Dwellings are classified according to the type (e.g., apartment, detached house) they belong to and the heating systems they are equipped with. The second method is a model trained for image recognition and classification based on machine learning techniques. The images from the real estate advertisements are collected and processed to identify wood burning installations, which are automatically classified according to the three classes used in official statistics, i.e., open fireplaces, stoves produced before 1998 and stoves produced after 1998. The model recognizes and classifies the wood appliances with a precision of 81%, 85% and 91% for open fireplaces, old stoves and new stoves, respectively. Emission factors are heavily dependent on technology and this information is therefore essential for determining accurate emissions. The collected data are compared with existing information from the statistical register at county and national level in Norway. The comparison shows good agreement for the proportion of residential heating systems between the webcrawled data and the official statistics. The high resolution and level of detail of the extracted data show the value of open data to improve emission inventories. With the increased amount and availability of data, the techniques presented here add significant value to emission accuracy and potential applications should also be considered across all emission sectors.

## Introduction

Methods for data collection are evolving following changes both in technology and society. With the development of information and communication technologies (ICT), new efficient techniques within both natural and social science are being developed, for instance social media analytic with environmental data [1]. Data collection is the initial and one of the crucial steps in the development of accurate emission inventories [2]. Therefore, there is an increasing need to develop and test new methodologies for data collection.

New methods open up new opportunities, such as the development of applications that provide information at high spatio-temporal resolution. This is of high interest for modeling air quality at the urban scale. Air quality models (AQMs) are an essential tool to support the decision-making process through evaluation of the possible impacts of local emission abatement policies on air quality and human health. To produce accurate results, AQMs rely on meteorology, boundary conditions and emission inventories as input data. The quality of the latter is critical in a polluted area, where local emissions contribute significantly to pollution levels, as typically occurs for most large cities and industrial areas. Authorities, and the public in general, desire finer resolution than what is available in most current AQMs that are run today, because coarse grid resolution models tend to underestimate exposure to pollutants [3]. Sub-grid parameterisations offer an attractive option for lowering model spatial resolution, but such methods are largely reliant on accurate spatial and temporal knowledge of emissions [4], and hence on accurate input data at high spatial resolution.

Emission inventories need to be developed according to methods that represent accurately the physical process of the emissions of interest. The approach selected depends very much on the input data availability, purpose of the study and the emission source intensity. At local scales, coarsely resolved emissions can be downscaled and distributed in space at finer resolution based on proxy data (e.g., population density and land cover data). Bottom-up methods can be used where sufficiently accurate activity data exists, it can be collected at fine spatial scale (e.g., building point, road link) and then aggregated. The latter are preferred methods, but the availability of input data at fine scales is still a challenge [5].

In our study, we focus on emissions from residential wood combustion (RWC). In Norway, as in other north European countries, RWC is the second most important source of domestic heating after electricity. There are around 2.5 million wood burning stoves in Norway, predominantly these are individual installations in living rooms, and over half of them are presumed to be in regular use. In Norway there is a strong tradition tied to wood burning, as it creates a cosy atmosphere and it is simple to operate. However, RWC is also a main contributor to harmful air pollutants (e.g. particulate matter, polycyclic aromatic hydrocarbons-PAHs) and an important source of black carbon, a short-lived climate pollutant [6]. Both the emission estimates and their spatial allocation are highly uncertain. Those are largely associated with the activity data, namely the wood consumption per household and the emission factors for the different technologies [7–9].

Norwegian national emissions from RWC are currently estimated based on statistical data at the county level on the amount of wood consumed and the type of technology (e.g. fireplace, closed wood stove). This information is collected by Statistics Norway (SSB) through self-reporting questionnaires (SSB Travel and Holiday Survey; [10]). Around 6000 persons are asked annually about their wood consumption and type of wood-based heating technology, and the data are thereafter used to derive values at county and national level. When determining emissions from RWC at city level, the input data requires high spatio-temporal resolution to capture the variability in the urban environment. However, we currently lack detailed information on where emissions occur, or which dwellings are provided with wood-based technology or other heating systems. These entail large uncertainties, and RWC is considered as the largest source of uncertainty regarding particulate matter in cities [11]. The development of new and improved emissions from RWC in urban areas is therefore a priority. In the new Norwegian emission model MetVed for RWC, high spatially resolved data is used as input data and, as a result, significant improvements of modelling results when compared with observations are observed [12].

The primary objective of our study was to develop a method for placing the geographical location of wood-based heating technologies for RWC and their classification. This allows us to establish the location of RWC emissions sources with a higher precision than currently applied methods, and to determine how emissions vary in the urban environment. We describe two methods for extracting open data and processing it with the aim of improving the input data for emission inventories from RWC. One of the methods is an open source webcrawler developed for the purpose of our study, i.e. GoodOvening (https://github.com/mattp/good_ovening), and applied as a method for extracting and analyzing data from a real estate advertisement portal. Webcrawling or scraping has been defined as the process of extracting and combining in a systematic way contents of interest from the Web. The webcrawling accesses the webs of interest, analyses the content in order to find and extract the needed data, and then structures them in a defined way for further data analysis [13]. We also present a trained model for the recognition and classification of the webcrawled images of wood-based technologies based on machine learning. This allows us to know with a high level of detail the distribution of RWC technologies and therefore their emissions.

Webcrawling has extensively been applied in data collection for different applications such as collecting air quality measurements and forecast from open web pages [14], identifying modern “traditional” medicine from user generated blogs [15], extracting information from social web forums [16], among others (see [17]). To our knowledge, it is the first time that webcrawling is used or reported in the literature as a method to collect spatial data for the development of high resolution emission inventories. Our study brings new opportunities for the improvement of the spatial distribution of atmospheric emissions, and especially for emission sectors where input data collection is still a challenge. Several emission inventories are developed based on global or national statistics due to the lack of input data at finer spatial resolution. These emissions show important inconsistencies when compared with local estimates based on higher resolved data [18]. The methods presented in our study can be developed further to contribute to make data collection an effective process targeting other emitting sectors such as road and off-road traffic or construction activities. For these sectors, input data such as detailed information about fleet composition, machinery type or driving conditions at urban scale are still lacking for cities and national aggregated statistics and distributions according to geographical features (e.g. land use) are being used with their subsequent limitations [19]. The use of webcrawling over identified webs with openly available data at urban scale will contribute to a better understanding of local activity and therefore also emissions. The described methods enable the design of a cost effective methodology to establish a well-structured database for data analysis and the update of urban emission inventories.

## Methods

The overall methodological framework builds on the webcrawler that extract open data and graphic material (i.e., pictures) from an advertisement portal that contains the main bulk of the real estate market in Norway. For each property for sale or rent, detailed information on the dwelling is provided on-line. Among all the information describing the property, the available heating system (e.g., heat pump, district heating, wood stove) and the type of dwelling (e.g., detached house, apartment, cabin) are the most relevant for our study. The webcrawler extracts two types of data; 1) the geographical location of dwelling with the specific characteristics of interest and 2) the pictures. The second method presented in this manuscript applies to the processing of the pictures. A model for image recognition based on machine learning was trained to classify each individual residential wood-based heating technology, which is an essential variable in order to select appropriate emission factors and estimate emissions.

### Keyword dataset

Keywords constitute the drivers of the systematic search, and comprise the basics for both the word recognition process and the data extraction. A series of keywords were therefore prepared, based on different criteria aiming at localizing relevant information on residential heating from real estate postings, i.e., dwellings with wood burning appliances or other heating technologies. The first set of keywords were selected in order to identify the different types of heating system (i.e., heat pump, district or central heating) and more specifically the location of wood-based technologies for RWC (i.e., old stove, modern stove, fireplace). For this, we established a keyword dataset consisting of 94 keywords that cover a wide range of heating systems. This keyword dataset contains synonyms commonly used in Norway to describe the wood-based technologies, and additional attributes (e.g., old, modern) that may be applied in listings.

The second keyword list was made in order to characterize the variability in the type of dwelling. The keywords for residential type were the same as the classification used in the advertisement and also in the official statistics (i.e., detached house, duplex, townhouse, and apartment). Previous studies have highlighted the importance of taking into account the type of dwelling when addressing RWC and its subsequent emissions. The reason is that wood consumption has been reported to differ between types of dwelling, and for instance, it seems to be higher in detached houses than in apartments [20, 21]. The extraction of data by the second keyword list were implemented at a later stage and so are not included for all the data collected.

### The GoodOvening webcrawler

The data of interest is defined by the keywords dataset and the corresponding geographical coordinates are collected via the custom designed webcrawler (i.e., GoodOvening; https://github.com/mattp/good_ovening) built using the open-source Scrapy webcrawling framework (http://scrapy.org). The GoodOvening run over the Norwegian advertisements website FINN (http://finn.no). This is the largest real estate portal that covers the vast majority of real estate transactions in Norway. Scrapy provides a convenient Application Programming Interface (API) for parameterizing and deploying an automated “bot” designed to systematically browse webpages and scrape them for specific content defined in the keyword dataset. The API provides a base Spider class with an exposed parse function that allows for simple parsing of HTML objects within the pages, as well as pattern matching the predefined keywords on the text within those objects. A set of allowed domain-names provides boundaries for the spider to traverse within by disallowing traversal outside of sub-pages of the given domains. A set of starting URLs sets the initial locations from which to begin searching. The predefined keywords dataset is initialized in a Scrapy Item object to store the scraped data, which can then be converted into either JSON or XML output format. A Scrapy runtime option saves this formatted data to the specified type of output file.

The GoodOvening webcrawler uses a single allowed domain, the main FINN page (http://m.finn.no). The set of initial URLs corresponds to the root page of each housing transaction type for which there are available listings (i.e., homes for sale, lettings, business rentals). From each root page, the webcrawler follows all available links on that page, extracts data from the listing, and then follows the next page link, if available. This process is repeated until no next page is found. The procedure for extracting data from an ad listing is as follows:

1. Extract the Scrapy response object corresponding to the ad page;
2. Pattern-match on the HTML object containing the ad ID and save it to a variable;
3. Pattern-match on the HTML object containing the latitude and longitude of the dwelling and save them to variables;
4. Pattern-match on all HTML objects corresponding to the description text of the listing. Retain any descriptions containing predefined keywords (i.e. “fireplace”, “wood stoves”, “district heating” etc.);
5. Pattern-match on all HTML objects containing the image URLs and captions for pictures of the dwelling. Retain any links where the image captions contain predefined keywords,
6. Append the scraped data to the XML output file containing the aggregation of all currently collected data;

Once the full XML output file has been compiled, the data is further processed in two ways; first, keywords mentions are extracted from the XML and stored in a SQLite database alongside the ad ID, URL, and geographical coordinates. Where there are mentions of multiple keywords (e.g., different stove-types, or stove and district heating) in the same advertisement, they are stored in separate rows; and second, a separate module extracts the saved image links and manually downloads them to disk in a directory named with the corresponding advertisement ID. The post-processing allows for fast querying of wood stove-type statistics and locations, as well as cross-referencing of wood stove-types with, presumably, images of the corresponding fireplace or stove.

The GoodOvening webcrawler is designed to be run once per day and the output XML file is stored by date stamp. However, duplicate entries of the same advertisement are not added to the database, as each advertisement has a unique ID and only new IDs are extracted, so if no new data has been added to the site, there will be nothing new to process. If an object has been listed several times under different IDs it will occur multiple times in the database. FINN allows for updates, renewal and changes of listings without service charge and under the same ID. Double entries of the same object will corresponds to new announcements, and as it is considerably more effort to make a new real estate entry rather than update an existing one, double entries are few over long time webcrawling. By manual inspection, multiple entries of the same address showed it was in fact different apartments in the same buildings.

### Object recognition by machine learning

A machine learning algorithm was trained to detect wood-based burning technologies for RWC in images collected during the GoodOvening webcrawling process. A pre-trained model of a convolutional neural network (CNN) https://pjreddie.com/darknet/yolo/ was used as a starting point for the training. The weights of the darknet framework [22] were used to run the YOLO v2 object detection model [23].

Specifically the algorithm was trained to recognize three different oven technology classes, for which emission factors of wood-based technologies are defined. A total of 4946 individual images of variable size were manually classified for training and testing. The three classes of wood-based technologies were equally distributed among the images. The pictures were re-sized and padded to a size of 1200 × 800 pixels. The images contain at least one of the oven categories of interest along with other objects commonly found in living rooms (e.g., furniture, windows, electronic equipment). Around 70% of the manually classified images were used during the training phase, the remaining were used for testing of the algorithm. Specifically, the batch was set to 64 with sub-batches of 32 and a decay of 1.0 × 10^−5^. The learning rate schedule was the following: 1.0 × 10^−4^ for the first 100 epochs. Then, 1.0 × 10^−3^ for the 1000 epochs and 1.0 × 10 − 4 for the rest. The threshold to consider a detection as a true positive during training was set to 0.6 (60% confidence).

## Results and discussion

### Data mining by webcrawling

**Fig 1:**
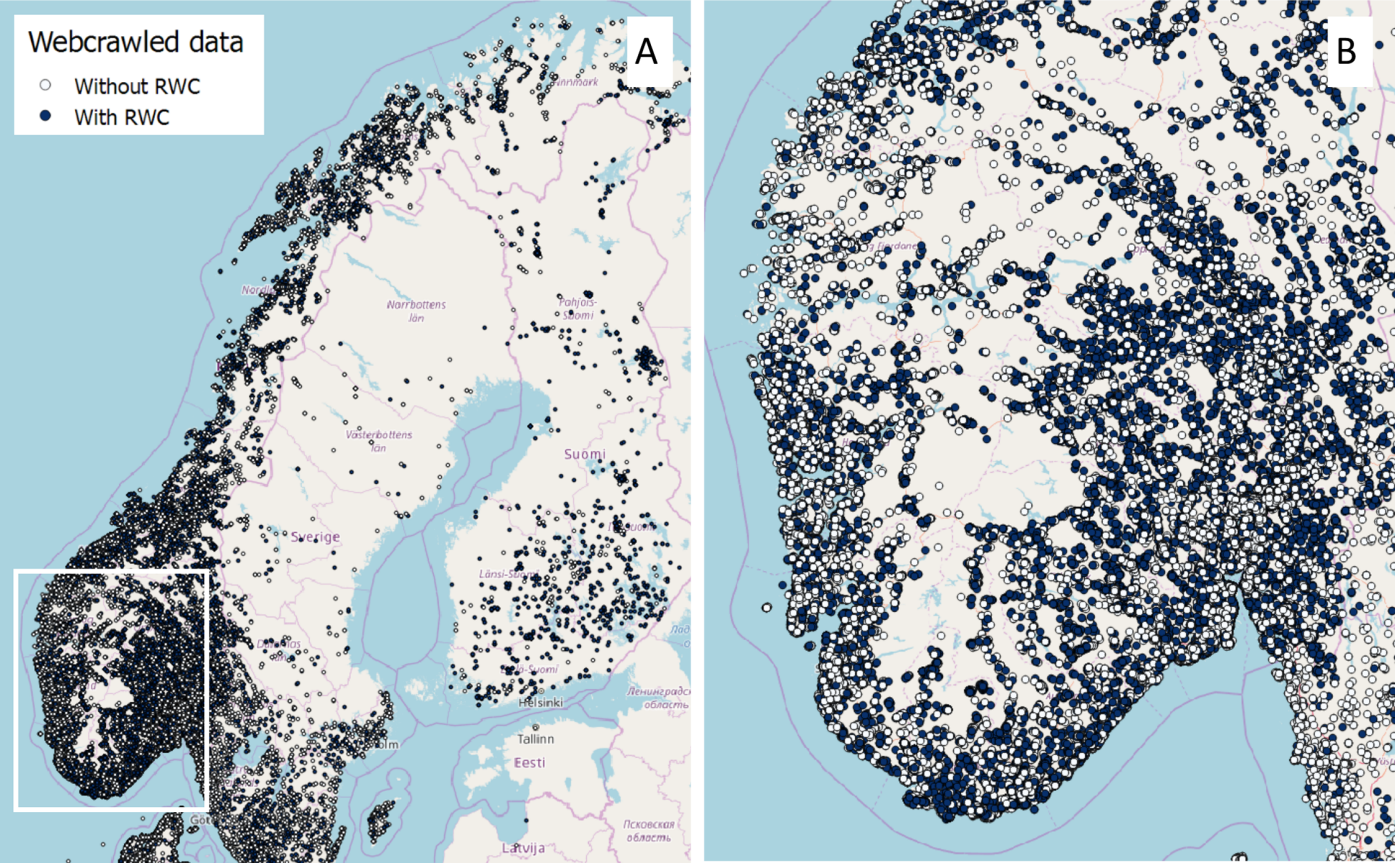
Geo-positioned data collected with the webcrawler. “Without RWC” refers to properties without residential wood combustion technology, and “With RWC” refers to properties with residential wood combustion technology. The rectangle in A corresponds to the area represented in the module B. Reprinted from [24] under a CC BY license, with permission from NILU - Norwegian Institute for Air Research, original copyright (2018), S1 Fig.

Around 437000 geo-positioned data points were crawled in Norway from the classified advertisement portal FINN based on the criteria defined by the keyword dataset. Fig 1 shows the webcrawled data and its geographical distribution. Each data point represents a real estate listing, containing information on individual listings. The data sampling covers both inside and outside Norway as FINN includes properties for rent or sales abroad. Dwellings for sale, rent and properties for holidays constitute 31, 32 and 26% of the total data sample, and the remaining consists of newly built or planned dwellings (2%), leisure for sale (4%), business for rent (1%), properties abroad (1%) and others. Preliminary, we evaluated the collected data within Norway per county to assess the geographical distribution. Four out of the 19 counties in Norway comprise 46% of the collected data. These counties include the largest and most populated cities in Norway (i.e., Oslo, Trondheim, Bergen and Stavanger) with about 23% of the Norwegian population. This is also where we found the highest turnover of housing properties for sales and/or rent, so data intensity is further amplified by this.

**Fig 2:**
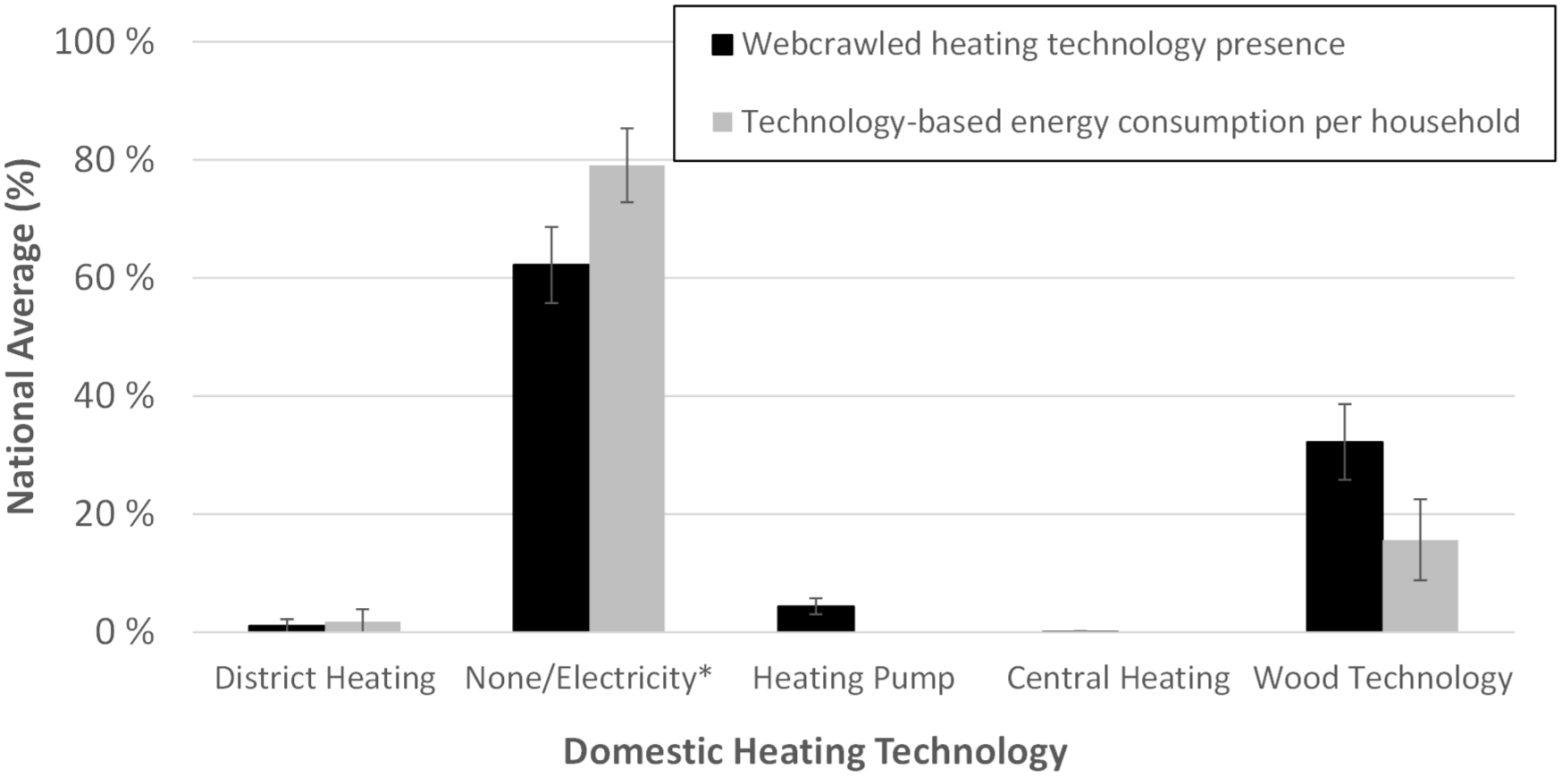
Average distribution of 1) domestic heating systems in the webcrawled sample and 2) technology-based energy consumption per household from official statistics [25]. The values represent national averages and the bars the standard deviation. * refers to “None” for the crawled sample, and “Electricity” for the domestic heating technology.

The extracting process includes data on available heating technology in the residential properties, covering both wood and non-wood based technologies (e.g., heat pumps, district heating, central heating). This constitutes an important piece of information to understand emissions from residential heating, as the availability of non-wood based technologies (e.g., district heating) can reduce the usage of RWC technologies in a particular property. The allocation of the different heating systems among the counties seems to show uniformity. Fig 2 shows with the black bars the average distribution of heating technologies in the webcrawled data, i.e., district heating, heat pump, central heating, wood based technology and none. The latter refers to dwellings that do not satisfy any of the keyword criteria. The unclassified listings account for around 60% of the data. For these we assume that electrical stoves are their main heating system. Energy saving heating systems such as central heating and heat pump are generally mentioned in listings as they increase the value of the property. Similarly, having a wood stove as an additional heat source is considered positive, hence the absence from the advertisement will in most cases be a strong indicator of its absence in reality. In addition, the grey bars in Fig 2 show the distribution of technology for energy consumption per household based on official statistics [25]. Electricity, including heat pumps and central heating, constitutes the primary energy consumption source in households in Norway, followed, in order of usage, by wood based fuel, oil and kerosene and, gas and district heating. The second most frequently webcrawled heating technology is wood based technology (around 30%). This relatively high share is in agreement with official statistics that establish wood as the second most important energy commodity in households (Fig 2). However, this comparison needs to be considered carefully as we are comparing the presence of the heating technology against the energy consumption per technology.

**Fig 3:**
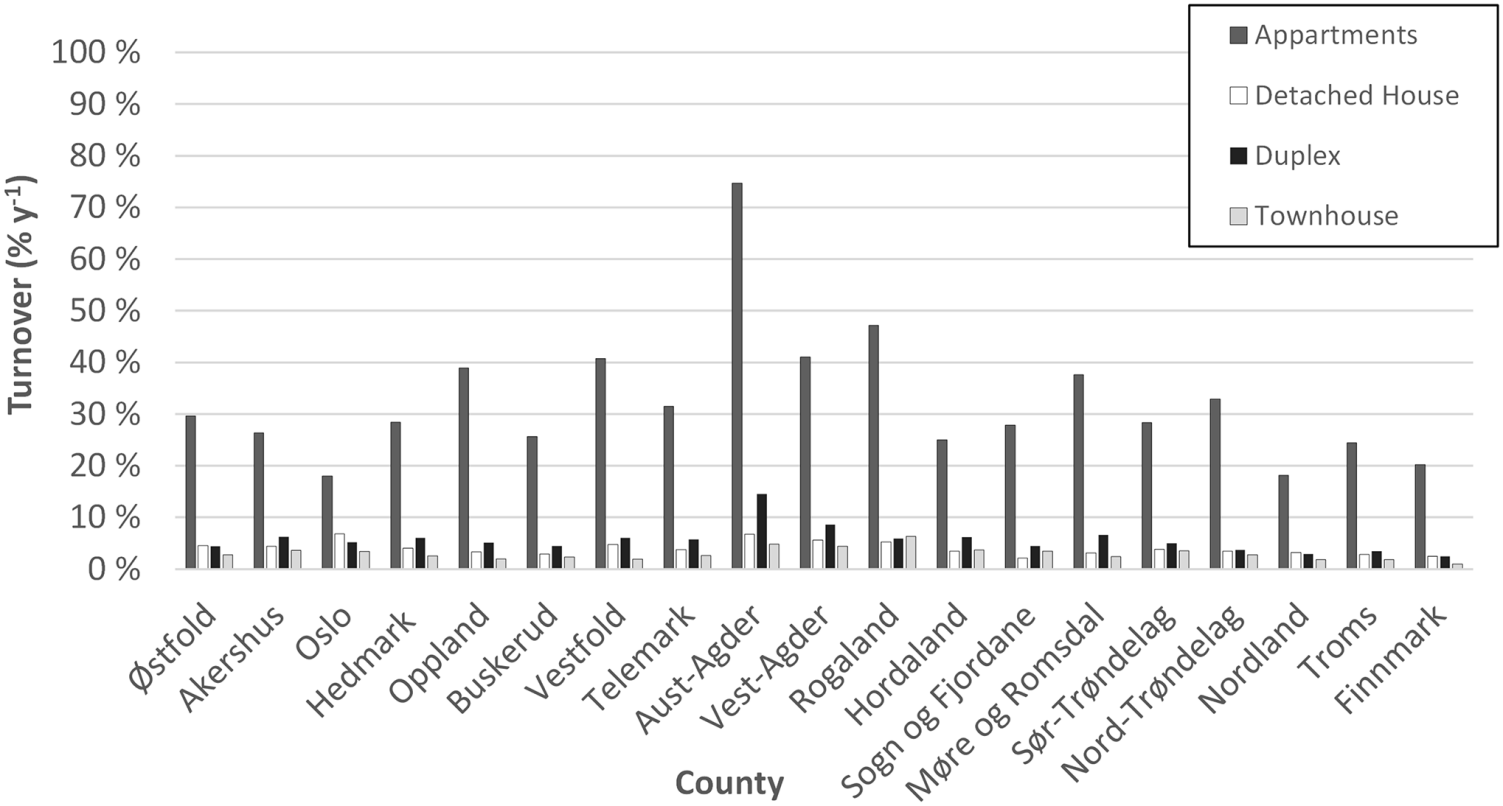
Turnover rate (% y-1) per county estimated from the webcrawled sampling.

The type of dwelling is an important variable when addressing heating technology. For instance, heat pumps are commonly used in detached houses, while apartments are more commonly connected to district heating. Some studies assume that wood burning is more prevalent in single houses than in apartments. Among the reasons given are that apartments have a lower heating demand, the wood storage and access is limited, and the transport of the wood is inconvenient [20]. In our study, we use the classification commonly used by Statistics Norway and also the one most frequently used in the real estate advertisements, hence dwellings are classified as apartments, detached houses, townhouses or duplexes. The latter three classes form all together around 70% of the Norwegian housing stock. Additional types of dwellings are also collected, such as cabins, but are not included in the evaluation in this manuscript. The highest number of crawled dwellings are apartments, and they make up a significantly higher share of the crawled data than they do of the national official dwelling numbers. This is due to a higher turnover rate of apartments than other types of dwellings as it is shown in Fig 3. The turnover of apartments is on average around 32%, whereas for the different types of houses is around 4%. The factor 8 difference in turnover rate between apartments and other dwellings leads to a sample bias that needs to be accounted for in our aggregated statistics. For instance, the webcrawling data suggest that there is a significantly lower probability that apartments have a small domestic wood-based heating system (19%) compared to the probability for the other types of dwellings (50%) at national level. This can be corrected for by treating dwelling categories separately. However, the potential biases within each category of dwelling are harder to account for.

In order to investigate some of the sampling biases that we might face we tested the webcrawled sample of dwelling types against an official database containing all residential buildings in Norway (i.e., the SSB database). We tested urban area residences against rural areas. For this, we classified an urban residential building in accordance with the Statistics Norway definition for urban settlements (see http://www.ssb.no/en/klass/klassifikasjoner/110). For the building types, we could observe the following distributions of urban and rural buildings. For detached houses, the webcrawling sample shows 73% in urban areas and 27% in rural areas, whereas for the SSB database the respective share is 45% and 55%, respectively. The distribution of duplexes in the webcrawled sample is 90% in urban areas and 10% in rural compared to the SSB database which shows 70% and 30% for the two areas. Townhouses and apartments have a similar distribution in the webcrawled sample with 95% in urban areas and 5% in rural, whereas apartments in the SSB database are distributed as 77% in urban areas and 23% in rural areas, and townhouses as 88% in urban areas and 12% in rural areas.

From this data evaluation we establish that urban properties are traded more frequently than rural properties, and thus represent a higher share of the traded market than their share of the total houses. To investigate if there is a significant difference between rural and urban properties, we looked at the frequency of a wood-based heating technology in these categories. We found that the probability of having a wood-based heating technology in a detached house in the webcrawled sample is 56% for urban areas, whereas for rural areas it is 54%. This indicates that there is not a significant difference in the frequency of wood-based technologies between urban and rural dwellings in Norway. Other housing characteristics, such as building age and size of the building may also influence how well buildings of such types are represented on the market, and so we are not able to rule out sample biases completely, though the parameters we could test show no indication of this.

**Fig 4:**
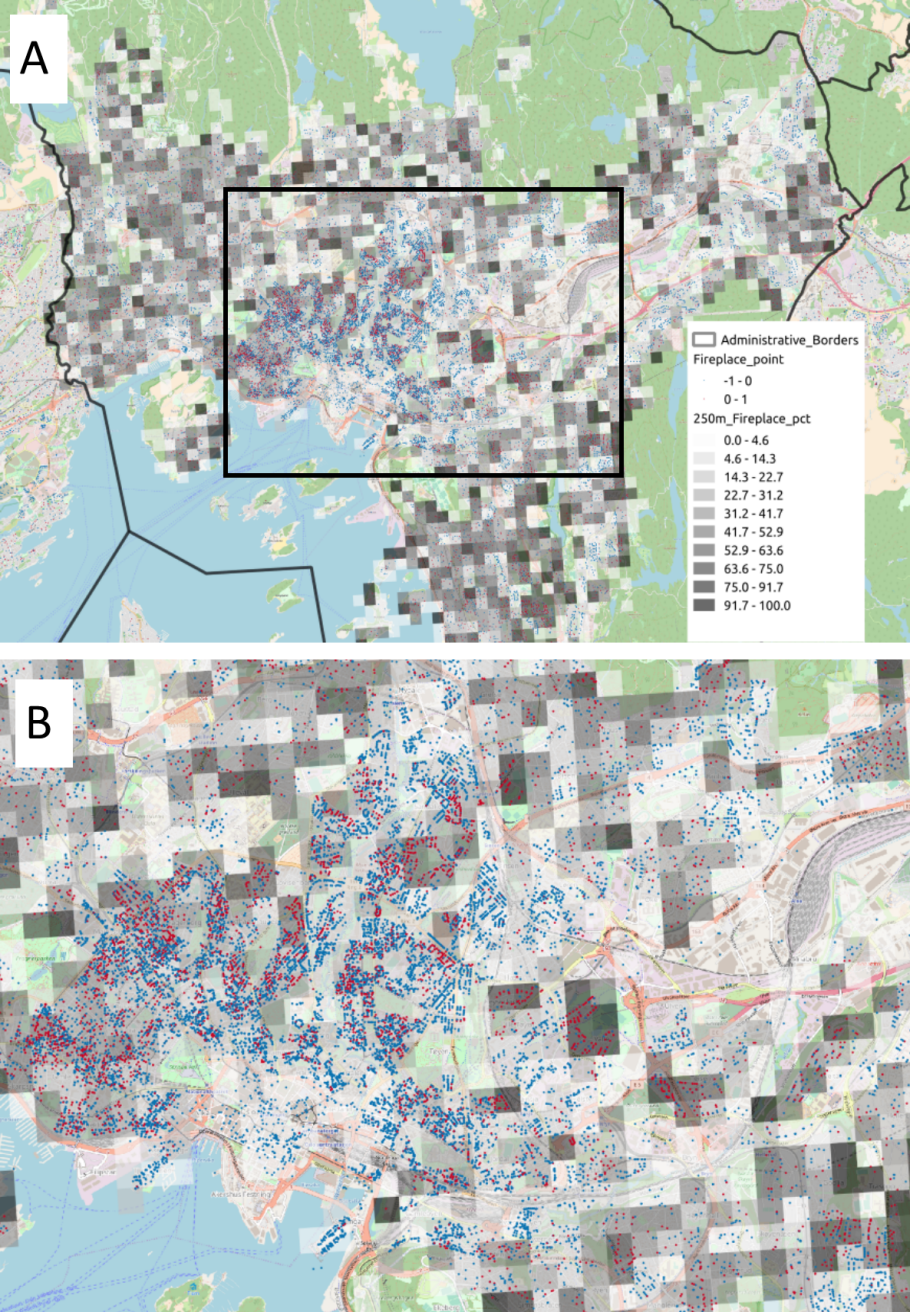
Webcrawled data in Oslo municipality. Red dots represent dwellings provided with wood-based technologies for residential heating, blue dots represent dwellings provided with a technology for residential heating different from wood-based technology. The grids represent the relative share of wood-based technologies regarding other heating systems. The rectangle corresponds to the area represented in the module B of the figure. Reprinted from [23] under a CC BY license, with permission from NILU - Norwegian Institute for Air Research, original copyright (2018), S2 Fig.

One of the advantages of the webcrawling is that the data is collected as geo-located points supporting the analysis at high resolution. The evaluation of the data contributes to understanding the distribution of proportions of residential heating technologies within Norwegian urban areas. With sufficient data points, correctly placing the existence of RWC within individual buildings, with their associated technology type will subsequently improve the spatial distribution of emissions associated to the dwelling. Fig 4 represents the webcrawled data in and around Oslo as points on a map. Dwellings with wood-combustion are shown as red dots, the dwellings without are shown as blue dots in Fig 4. The greyscale grids have a resolution of 250 m and represent the fraction of points within the grid that have a wood burning installation. The total sample density increases towards the more densely populated city centre. We observe that the highest density of wood-based technologies appears to be around downtown Oslo, matching the highest density of dwellings. However, as it is shown by the grayscale map, where the density of RWC technologies seems to be high, we also obtain the lowest proportion of dwellings with a RWC technology (Fig 4). One implication of this is that a simple scaling down by proxy of, e.g., dwelling density would over-estimate emissions in densely populated areas. In Oslo downtown we have a high number of apartments connected to district heating or using electricity as their exclusive heating commodity. This can explain the low share of wood-based technologies in this area. Previous studies establish that wood consumption is lower when other heating technologies such as district heating are also available, e.g., [14]. Our results contrast with methods used to spatially distribute emissions from residential heating based on proxy data such as population or dwelling density [4], [26], and it supports the concern that such methods may over-allocate emissions in highly populated areas [5].

### Image recognition and classification

**Fig 5:**
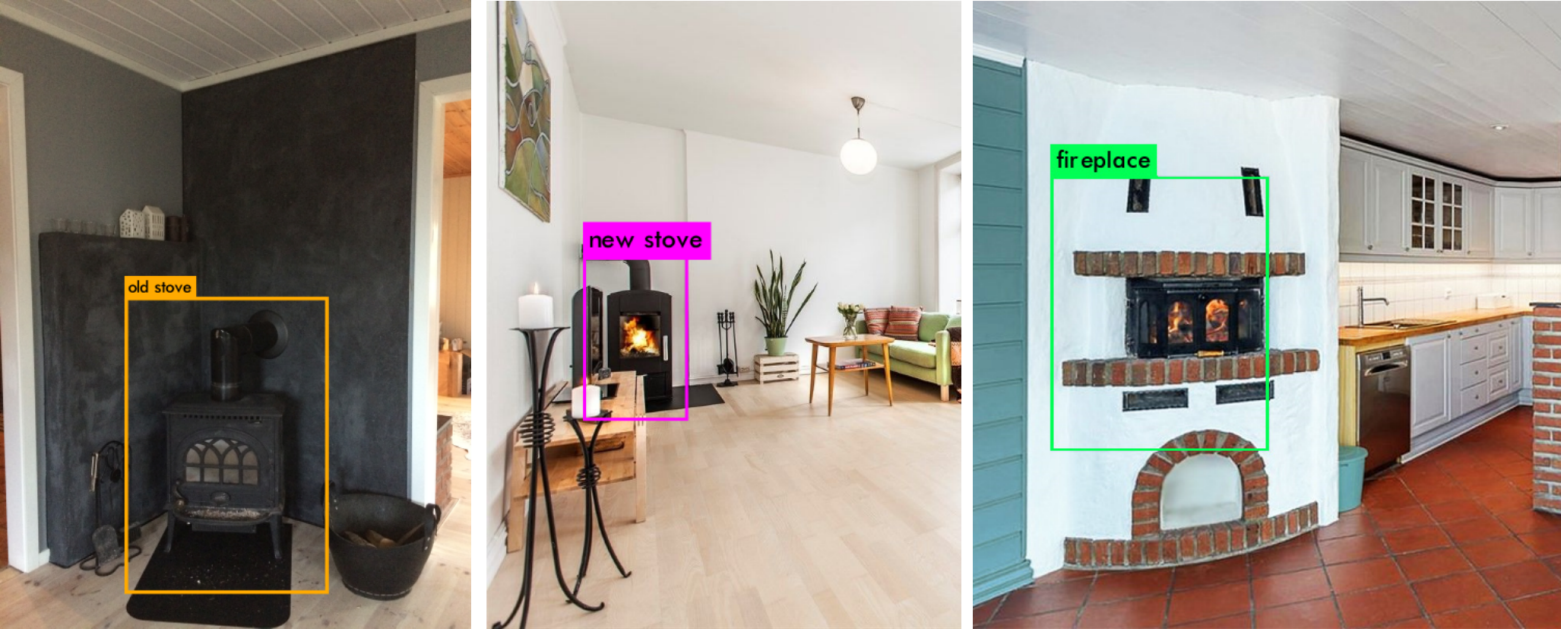
Example of image recognition and classification of wood-based heating technology for fireplaces, new stoves and old stoves.

A validation set of 1488 images were used to measure the performance of the algorithm. Precision, recall and mean average precision (*mAP*) were used as metrics for the trained model. Precision refers to the percentage of objects that were classified correctly. By correct classification, we mean as compared to the one carried out manually by a person and based on the physical features of the technology, i.e., an open fireplace versus a stove, and old versus modern aesthetics. The recall is the ratio of correctly predicted positive values to the actual number of positive values. The average precision was computed separately for each class and then averaged for all of the classes. A detection was consider a true positive when the intersection over union (*IoU*) with a ground-truth box was greater than 0.6. The average recall was estimated in 91.5% while the *mAP* was 85.7%. Table 1 shows the average precision for each of the categories, where the precision was the highest for new stoves, followed by old stoves and fireplaces. Fig 5 shows an example of the image recognition performance and the classification of wood-based technologies for the three selected categories. The algorithm is able to identify and correctly classify objects that are only partially represented in the pictures, as the new stoves shown in Fig 5.

**Table 1:** Average detection precision for the wood burning technologies.

Technology Class	Average Precision (%)
new stove	91.3
old stove	84.9
fireplace	80.8

**Fig 6:**
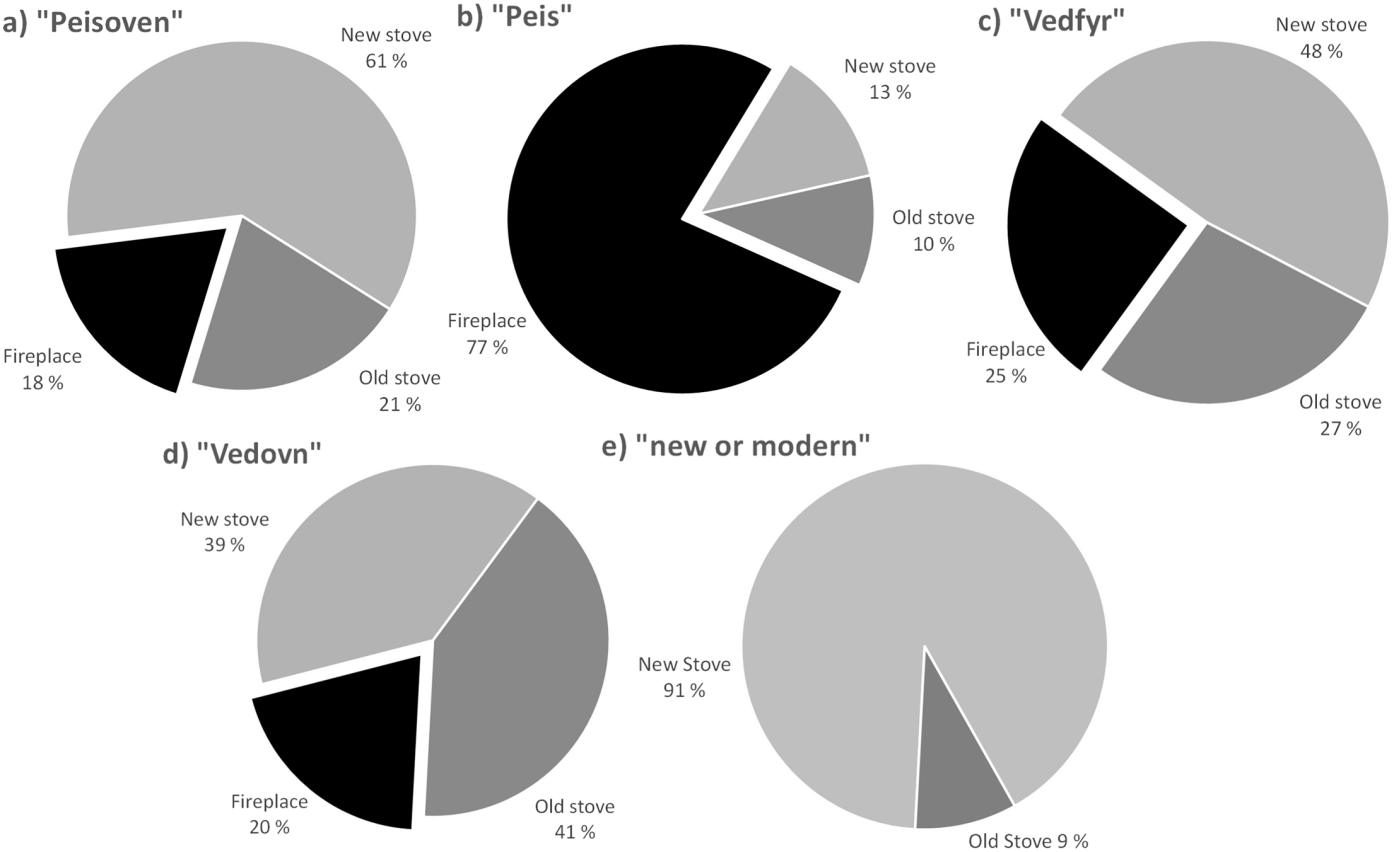
Comparison of the most frequent webcrawled keywords (“Peisoven”, “Peis”, “Vedfyr” and “Vedovn”) and the attribute “new or modern” and the corresponding picture classification (Fireplace, New Stove, Old Stove).

One of the objectives of developing the machine learning routine for classifying images on different technology classes is to supplement the information obtained by the webcrawled data. Determining the type of wood-based technology is crucial for understanding and developing the emission inventory because emission factors are very much dependent on the technology type. The keyword dataset developed for the GoodOvening webcrawler contains around 80 keywords referring only to wood-based technology, including the combination with different attributes (e.g., modern, old). The keywords are not used systematically by the public to refer to the different types of burning appliances, e.g., the same wood-based technology is named in different ways, and the same word is used to refer to different technologies. As a consequence, an accurate classification of technologies is not possible just based on the webcrawled data. In order to assess a potentially systematical use of keywords referring to specific technologies, we compared the results from the automatic classification of pictures with the keywords contained in the corresponding advertisements. This is done over a sample of around 27000 pictures that is built with the images collected from the advertisements that contain the most frequently used keywords, i.e., “vedovn”, “peis”, “peisovn” and “vedfyring”. The pictures are classified by our model as fireplace, new stove, and old stove, and the results are compared with the words used in the advertisements aiming at finding their potentially systematic use (Fig 6). The comparison indicates that the word “peis” is commonly use to refer to open fireplaces in the real state advertisements whereas “vedovn” refers commonly to closed stoves. The words “peisoven” and “vedfyr” seem to be used inconsistently to refer to different wood-based technologies. The lowest frequency of fireplaces, 18% and 25% for “peisoven” and “vedfyr”, respectively, may represent the lowest occurrence of this technology class in Norway compared to closed stoves, which constitute around 95% of the wood-based technologies for residential heating. A similar evaluation was carried out over the advertisements that contains the attributes “new” or “modern” when describing wood burning stoves, and in this case 91% of the images are classified as new stoves by our model (Fig 6).

## Conclusions

We developed an open source webcrawler for extracting and classifying geo-located data and images of dwellings and their heating technologies from a real estate advertisement portal. The method is moreover complemented with a trained model for the automatic classification of wood-based technologies for residential heating based on image recognition by machine learning. Our study shows the usefulness of webcrawling as a data extract method for data analysis, and especially for applications that require information at high spatial resolution.

The study was carried out with the overall aim of improving the geographical distribution of emissions from residential wood combustion, an important air pollution source in several urban areas whose characterization encompasses significant challenges. Our study shows a large potential of such methods for gathering and analyzing input data at a high level of detail. The assessment of the spatial distribution of residential heating technologies shows that, even thought the highest density of wood-based technologies match the highest population or dwelling density, their share regarding other heating technologies is the lowest. This largely contributes to the knowledge of the spatial distribution of wood consumption for residential heating, and to the improvement of the emissions associated with.

The assessment of the collected data and its comparison with official statistics supports the use of the method, and especially of the combination of this data with other existing databases to reduce possible bias. The methods presented in this study have a strong potential to improve information for other emitting sectors, where data availability constitutes one of the biggest challenges and main sources of uncertainty.

## Supporting information

S1 Fig Webcrawling database. Reprinted from [24] under a CC BY license, with permission from NILU - Norwegian Institute for Air Research, original copyright (2018).
(PDF)

S2 Fig Webcrawled data in Oslo municipality. Reprinted from [24] under a CC BY license, with permission from NILU - Norwegian Institute for Air Research, original copyright (2018).
(PDF)

## References

[1] Cerquitelli T, Quercia D, Pasquale F. Transparent Data Mining for Big and Small Data Studies in Big Data. Switzerland: Springer Nature; 2017.

[2] Houyoux MR, Vukovich JM, Coats CJ, Wheeler NJM, Kasibhatla PS. Seasonal Model for Regional Air Quality (SMRAQ) project. J Geophys Res. 2000;105:9079–9090. 10.1029/1999JD900975

[3] Li Y, Henze DK, Jack D. Kinney PL. The influence of air quality model resolution on health impact assessment for fine particulate matter and its components. Air Qual Atmos Health. 2016;9:51–68. 10.1007/s11869-015-0321-z 28659994 PMC5484574

[4] Kuenen JJP, Visschedijk AJH, Jozwicka M, Denier van der Gon HAC. TNO-MACC II emission inventory; a multi-year (2003–2009) consistent high-resolution European emission inventory for air quality modelling. Atmos Chem Phys. 2014;14:10963–10976. 10.5194/acp-14-10963-2014

[5] López-Aparicio S, Guevara M, Thunis P, Cuvelier K, Tarrasón L. Assessment of discrepancies between bottom-up and regional emission inventories in Norwegian urban areas. Atmos Environ. 2017;154:285–296. 10.1016/j.atmosenv.2017.02.004

[6] Bond TC, Doherty SJ, Fahey DW, Forster PM, Berntsen T, DeAngelo BJ, et al Bounding the role of black carbon in the climate system: A scientific assessment. J Geophys Res Atmos. 2013;118:5380–5552. 10.1002/jgrd.50171

[7] Seljeskog M, Goile F, Sevault A, Lamberg H. Particle emission factors for wood stove firing in Norway. Trondheim: SINTEF Energi AS; 2013.

[8] Evangeliou N, Shevchenko VP, Yttri KE, Eckhardt S, Sollum E, Pokrovsky OS, et al Origin of elemental carbon in snow from western Siberia and northwestern European Russia during winter–spring 2014, 2015 and 2016. Atmos Chem Phys. 2018;18:963–977. 10.5194/acp-18-963-2018

[9] Kindbom K, Mawdsley I, Nielsen OK, Saarinen K, Jónsson K, Aasestad K. Emission factors for SLCP emissions from residential wood combustion in the Nordic countries. Improved emission inventories of Short Lived Climate Pollutants (SLCP) [Internet]. TemaNord 2017:570. Rosendahls: Nordic Council of Ministers; 2018. Available from: https://www.diva-portal.org/smash/get/diva2:1174670/FULLTEXT01.pdf

[10] Norwegian Environment Agency, 2014. Informative Inventory Report (IIR) 2014. Norway. Air Pollutant Emissions 1980-2012 [Internet]. Oslo: The Norwegian Environment Agency. Available from: http://www.miljodirektoratet.no/Documents/publikasjoner/M125/M125.pdf

[11] Tarrasón L, Sousa Santos G, Vo Thanh D, Vogt M, López-Aparicio S, Denby B, et al. Air quality in Norwegian cities in 2015. Evaluation Report for NBV Main Results. Kjeller: NILU - Norwegian Institute for Air Research; 2017. Report No.: 21/2017. Sponsored by the Norwegian Environment Agency.

[12] Grythe H, Vogt M, Lopez-Aparicio S. Method for development of high-resolution emissions from residential wood combustion. In: Sokhi R S, Gállego M J, Raj Tiwari P, Craviotto Arnau J M, Castells Guiu C, Singh V, editors. Proceedings of Abstract of the 11th International Conference on Air Quality, Science and Application; 2018 March 12-15; Barcelona: MANNERS; 2018.

[13] Glez-Peña D, Lourenco A, Lopez-Fernandez H, Reboiro-Jato M, Fdez-Riverola F. Web Scraping technologies in an API world. Brief Bioinform. 2013;15:788–797. 10.1093/bib/bbt026 23632294

[14] Tsikrika T, Moumtzidou A, Vrochidis S, Kompatsiaris I. Focussed Crawling of Environmental Web Resources: A Pilot Study on the Combination of Multimedia Evidence. In: Vrochidis S, Karatzas K, Karpinnen A, Joly A, editors. Proceedings of the International Workshop on Environmental Multimedia Retrieval (EMR 2014). Glasgow, UK; 2014.

[15] Helfenstein A, Tammela P. Analyzing user-generated online content for drug discovery: Development and use of MedCrawler. Bioinformatics. 2017;33(8):1205–1209. 10.1093/bioinformatics/btw782 28011767

[16] Audeh B, Beigbeder M, Zimmermann A, Jaillon P, Bousquet C. Vigi4Med Scraper: A Framework for Web Forum Structured Data Extraction and Semantic Representation. PLoS ONE. 2017;12(1):e0169658 10.1371/journal.pone.0169658 28122056 PMC5266266

[17] Xu S, Yoon H-J, Tourassi G. A user-oriented web crawler for selectively acquiring online content in e-health research. Bioinformatics. 2014;30:104–114. 10.1093/bioinformatics/btt571 24078710 PMC3866553

[18] Sheng JX, Jacob DJ, Maasakkers JD, Sulprizio MP, Zavala-Araiza D, Hamburg SP. A high-resolution (0.1° × 0.1°) inventory of methane emissions from Canadian and Mexican oil and gas systems. Atmos Environ. 2017;158:211–215. 10.1016/j.atmosenv.2017.02.036

[19] Maes J, Vliegen J, van de Vel K, Janssen S, Deutsch F, De Ridder K, et al Spatial surrogates for the disaggregation of CORINAIR emission inventories. Atmos Environ. 2009;43:1246–1254. 10.1016/j.atmosenv.2008.11.040

[20] Plejdrup MS, Nielsen O-K, Brandt J. Spatial emission modelling for residential wood combustion in Denmark. Atmos Environ. 2016;144:389–396. 10.1016/j.atmosenv.2016.09.013

[21] López-Aparicio S, Vogt M, Schneider P, Kahila-Tani M, Broberg A. Public participation GIS for improving wood burning emissions from residential heating and urban environmental management. J Environ Manage. 2017;191:179–188. 10.1016/j.jenvman.2017.01.018 28092754

[22] Redmon J. Darknet: Open source neural networks in C. c2013-2016. Available from http://pjreddie.com/darknet/

[23] Redmon J, Farhadi A. Yolo9000: Better, faster, stronger. In: Computer Vision and Pattern Recognition (CVPR): Proceedings of the 2017 IEEE Conference: 2017 Jul 21-26; Honolulu, USA. Danvers: The Institute of Electrical and Electronics Engineers, Inc; 2017. p. 6517–6525.

[24] Lopez-Aparicio S. Residential Wood Combustion in the Nordic Area—The need for high resolution emission inventories. Presentation at “ClairCity Public Meeting”. 2018 [Internet]. Available from http://iresponse-rri.com/results

[25] Statistics Norway, Energy consumption per households [Internet]. Available from: https://www.ssb.no/en/husenergi/

[26] Terrenoire E, Bessagnet B, Rouil L, Tognet F, Pirovano G, Letinois L, et al High-resolution air quality simulation over Europe with the chemistry transport model CHIMERE. Geosci Model Dev. 2015;8:21–42. 10.5194/gmd-8-21-2015

