# Supplementary material for: Webcrawling and machine learning as a new approach for the spatial distribution of atmospheric emissions
Source: PLoS One. 2018 Jul 16;13(7):e0200650. doi: 10.1371/journal.pone.0200650 (PMC6047804; doi:10.1371/journal.pone.0200650)

Data availability as the key

- **Dwelling number and type (g)**; 2.4 MM dwelling + cabins (*SSB*);
- **Energy consumption statistics (k)**; total and per energy sector; type of dwelling; 660k dwelling (*ENOVA*);
- **Fireplace / stoves locations (p/k)**; location of fireplace/stoves; model/type technology (Rentbrennende); >1000k locations / 101 municipalities (*Fire Departments*)
- **Webcrawling database (p)**; type of dwelling; heating source; >400k dwelling (*finn.no*);

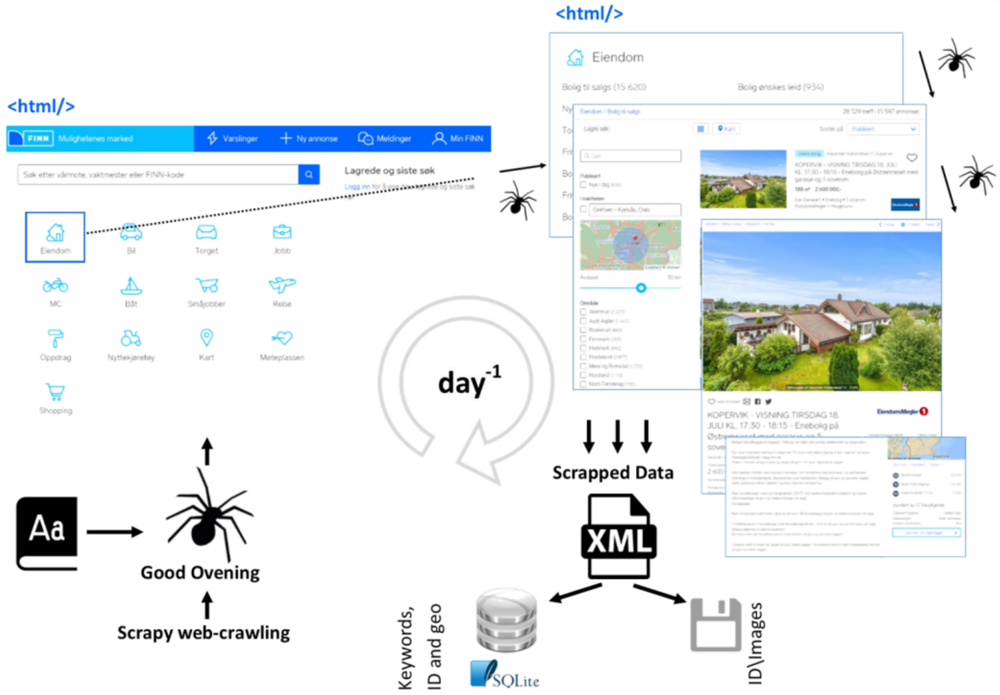

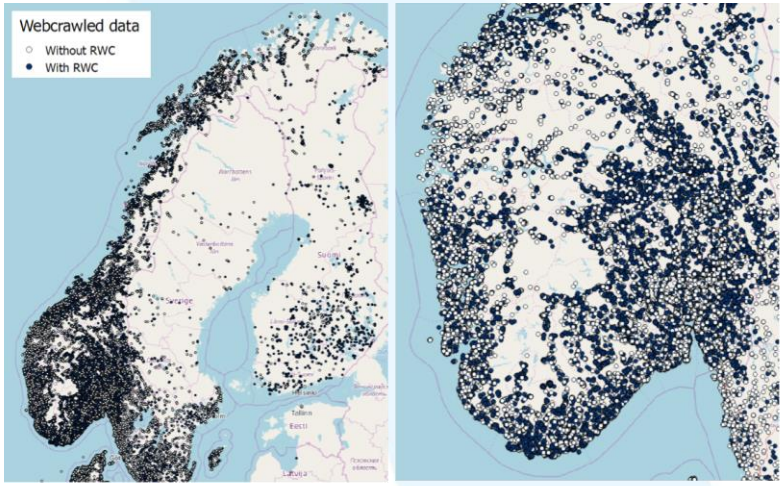

Supplement: S1 Fig — Reprinted from [24] under a CC BY license, with permission from NILU - Norwegian Institute for Air Research, original copyright (2018). (PDF) [file pone.0200650.s001.pdf]
